# Comprehensive analysis of *Panax ginseng* root transcriptomes

**DOI:** 10.1186/s12870-015-0527-0

**Published:** 2015-06-12

**Authors:** Murukarthick Jayakodi, Sang-Choon Lee, Yun Sun Lee, Hyun-Seung Park, Nam-Hoon Kim, Woojong Jang, Hyun Oh Lee, Ho Jun Joh, Tae-Jin Yang

**Affiliations:** Department of Plant Science, Plant Genomics and Breeding Institute, Research Institute for Agriculture and Life Sciences, College of Agriculture and Life Sciences, Seoul National University, Seoul, 151-921 Republic of Korea; Crop Biotechnology Institute/GreenBio Science and Technology, Seoul National University, Pyeongchang, 232-916 Republic of Korea

**Keywords:** *Panax ginseng*, Root, Transcriptomics, Ginsenoside, RNA-seq

## Abstract

**Background:**

Korean ginseng *(Panax ginseng* C.A. Meyer) is a highly effective medicinal plant containing ginsenosides with various pharmacological activities, whose roots are produced commercially for crude drugs.

**Results:**

Here, we used the Illumina platform to generate over 232 million RNA sequencing reads from four root samples, including whole roots from one-year-old plants and three types of root tissue from six-year-old plants (i.e., main root bodies, rhizomes, and lateral roots). Through *de novo* assembly and reference-assisted selection, we obtained a non-redundant unigene set consisting of 55,949 transcripts with an average length of 1,250 bp. Among transcripts in the unigene set, 94 % were functionally annotated via similarity searches against protein databases. Approximately 28.6 % of the transcripts represent novel gene sequences that have not previously been reported for *P. ginseng.* Digital expression profiling revealed 364 genes showing differential expression patterns among the four root samples. Additionally, 32 were uniquely expressed in one-year-old roots, while seven were uniquely expressed in six-year-old root tissues. We identified 38 transcripts encoding enzymes involved in ginsenoside biosynthesis pathways and 189 encoding UDP-glycosyltransferases.

**Conclusion:**

Our analysis provides new insights into the role of the root transcriptome in development and secondary metabolite biosynthesis in *P. ginseng*.

**Electronic supplementary material:**

The online version of this article (doi:10.1186/s12870-015-0527-0) contains supplementary material, which is available to authorized users.

## Background

Korean ginseng (*Panax ginseng* C.A. Meyer), a member of the Araliaceae family, is a tetraploid plant (2n = 4x = 48) whose haploid genome equivalent is greater than 3.5 gigabases [1, 2]. Ginseng is one of the most important medicinal crops, especially in East Asia. Triterpene saponins (referred to as ginsenosides), the principle bioactive compounds in *P. ginseng*, are biosynthesized via the isoprenoid pathway [3]. To date, more than 40 naturally occurring ginsenosides have been isolated from ginseng [4]. There are two main classes of ginsenosides (based on the skeletons of their aglycones), namely, the dammarane type and the oleanane type. Due to these ginsenosides, ginseng is widely used in traditional medicine and has a variety of pharmacological and physiological effects on humans, including anti-cancer, antidiabetic, immunomodulatory, neuroprotective, radioprotective, antiamnestic, and anti-stress activity [5–9].

Because of its commercial and medicinal importance, various genetic and genomic studies of *P. ginseng* have been performed [1, 2, 10, 11]. In recent years, next generation sequencing (NGS) technologies have been used to develop markers [11–13] and to identify several candidate genes encoding putative enzymes involved in ginsenoside biosynthesis in *P. ginseng* [14–18]. Although ginsenosides are biosynthesized in most ginseng tissues, including leaves, and berries, roots have one of the highest contents of ginsenosides and have therefore been used as a main ingredient in traditional medicines for over 2,000 years [6, 9, 19]. Accordingly, ginseng roots have been the focus of intense research [20] and have been increasingly produced throughout the world [21]. Traditional sequencing was initially used to obtain root expressed sequence tags (ESTs) of *P. ginseng* [22]. Since the emergence of NGS techniques, more than 80,000 ESTs have been deposited into the transcriptome shotgun assembly (TSA) database [14, 15]. However, no studies investigating deep sequencing of the root transcriptome (including ginsenoside biosynthesis genes) have previously been reported.

In this study, we generated a huge collection of RNA reads from different tissues and ages of *P. ginseng* roots using the Illumina sequencing platform. We applied a novel approach to enable the unigene set to be utilized for efficient downstream analysis and reliable interpretation. We also performed comprehensive characterization of the root transcriptome and expression profiling of important transcripts such as those involved in ginsenoside biosynthesis. The transcriptome data generated in this study provides new insights into the development of *P. ginseng* roots, as well as ginsenoside biosynthesis. Overall, comprehensive transcriptome data from various root samples of *P. ginseng* will serve as a valuable resource for discovering new genes related to root development and major secondary metabolite biosynthesis.

## Results

### *De novo* assembly of *P. ginseng* root RNA reads

Transcriptome sequencing of three independent biological samples of four root tissues yielded a total of 262,151,698 raw RNA-seq reads from pair of 131,075,849 reads (Table 1). All raw sequencing reads were deposited into the sequencing read archive (SRA) of NCBI (accession numbers SRR1648364, SRR1649308, and SRR1649311 for three replicates of one-year-old whole roots; SRR1648377, SRR1649321, and SRR1649325 for three replicates of lateral roots; SRR1648366, SRR1649315, and SRR1649319 for three replicates of main root bodies; SRR1648380, SRR1649327, and SRR1649331 for three replicates of rhizomes). After stringent quality checks and removal of adaptor sequences, 244,192,680 high-quality reads with base quality scores greater than 25 were obtained (Table 1). Additionally, we included reported RNA-seq data from ChP adventitious roots [17] for assembly to increase the coverage. Initially, a total of 486,622 transcripts were assembled using the Trinity *de novo* assembler.

**Table 1:** Summary of *P. ginseng* root transcriptome data acquired in this study

Sample	Raw		After filtering	
	No. of reads	Length (bp)	No. of reads	Length (bp)
One-year-old roots				
Whole roots, replicate 1	33,390,674	3,372,458,074	32,177,717	3,517,631,636
Whole roots, replicate 2	14,967,974	2,087,854,507	13,474,584	1,873,238,319
Whole roots, replicate 3	15,363,222	2,152,050,739	13,768,020	1,922,229,967
Six-year-old roots				
Main body, replicate 1	33,991,964	3,433,188,364	32,893,508	3,247,044,556
Main body, replicate 2	12,654,102	1,760,838,610	11,308,086	1,567,923,239
Main body, replicate 3	16,326,086	2,265,681,167	14,617,400	2,020,797,690
Lateral roots, replicate 1	33,938,598	3,427,798,398	32,883,593	3,248,608,642
Lateral roots, replicate 2	18,047,934	2,513,373,878	16,017,038	2,221,382,191
Lateral roots, replicate 3	16,166,344	2,261,990,497	14,277,362	2,261,990,497
Rhizomes, replicate 1	34,968,250	3,531,793,250	33,898,134	3,350,538,852
Rhizomes, replicate 2	17,900,886	2,505,960,830	15,937,196	2,222,852,715
Rhizomes, replicate 3	14,435,664	2,012,659,529	12,940,042	1,797,688,838
*In vitro* cultured roots				
Adventitious roots [17]	90,242,024	9,114,444,424	85,335,736	8,441,707,472
Total	352,393,722	40,440,092,267	329,528,416	37,693,634,614

In most previous transcriptome studies in *P. ginseng*, other types of housekeeping and regulatory RNAs were not eliminated from the assembled transcripts. However, initial *de novo* assembled unigene sets include other types of RNA such as rRNA, long-noncoding RNA (lncRNA), and even sequences from microbial organisms, which make downstream analyses difficult. Hence, we removed these unnecessary sequences, accounting for approximately 52 % of the initial transcript set, ultimately obtaining a total of 232,702 contig sequences (including isoforms) for *P. ginseng,* with an average length of 1,752 bp and a maximum length of 20,589 bp (Table 2). Subsequent downstream analysis of sequences including isoforms would reduce the accuracy of biological interpretations. Therefore, we generated an Nr unigene set without isoforms for further downstream analysis using a new strategy (Additional file 1: Figure S1). Over 95 % of filtered transcripts aligned to ginseng genome scaffold sequences [23], resulting in the identification of 44,665 corresponding gene loci. The remaining 5 % of filtered transcripts did not align to the scaffold sequences and represented 11,284 unique (without isoforms) Trinity assembly components. Finally, through selection of consensus exon sequences based on length, we obtained a total of 55,949 transcripts comprising our Nr unigene set (Table 2), which was used for all subsequent analyses. The transcript length in the Nr unigene set ranged from 201 to 20,589 bp, with an average length of 1,250 bp (Table 2), and most transcripts were shorter than 2 kb (Additional file 2: Figure S2). Among the 55,949 Nr unigenes, 39,381 transcripts matched 89.13 % of the 67,786 *P. ginseng* unigenes currently deposited in the TSA database (based on a homology search with a cutoff e-value of 10^−5^). Similarly, 19,608 Nr transcripts showed significant similarity to 92.9 % of the 17,773 reported *P. ginseng* ESTs in NCBI dbEST (as of September 2014). A total of 16,019 (28.6 %) transcripts were found to be novel sequences not previously generated or deposited into public databases for *P. ginseng* (Additional file 3. Dataset S1; http://im-crop.snu.ac.kr/transdb/data.php). Furthermore, we estimated the ratio of full-length open read frame (ORF) sequences using TransDecoder (included in the Trinity package), with the encoded protein length set to a minimum of 100 amino acids and homology search with swiss-prot and pfam databases. In all, 76.35 % of Nr unigenes were likely to be protein-coding sequences, of which 60.36 %, 18.53 %, and 9.73 % full-length, 5′ partial were, and 3′ partial sequences, respectively. The remaining 11.38 % of Nr transcripts were truncated (partial at both the 5′ and 3′ ends) or too short to predict their ORFs.

**Table 2:** Unigene set and functional annotation of *P. ginseng* root transcriptome

Unigene assembly	
No. of transcripts in initial assembly	486,622
No. of transcripts in filtered assembly	232,702
No. of transcripts in Nr unigene set	55,949
Max sequence length (bp)	20,589
Average sequence length (bp)	1,250
N50 length (bp)	1,998
Functional annotation	
Protein database	% of Nr unigenes
NCBI Nr	94.0
Swiss-Prot	75.6
TAIR (*A. thaliana*)	80.6
Tomato (*S. lycopersicum*)	84.5
Potato (*S. tuberosum* L.)	81.8

### Functional annotation of unigenes

To investigate the functions of the Nr unigene set transcripts, we conducted a sequence similarity search against Nr protein databases including NCBI, TAIR (*Arabidopsis thaliana*), Swiss-Prot, tomato (*Solanum lycopersicum*), and potato (*Solanum tuberosum* L*.)* using BLASTX (cutoff E-value < 1e^−5^). The results indicate that 94 %, 75 %, and 80 % of transcripts shared significant similarity with protein sequences in the NCBI Nr, Swiss-Prot, and TAIR database, respectively (Table 2; http://im-crop.snu.ac.kr/transdb/data.php). In addition, 84 % and 81 % of transcripts were also significantly similar to protein sequences in the tomato and potato database, respectively.

**Fig. 1:**
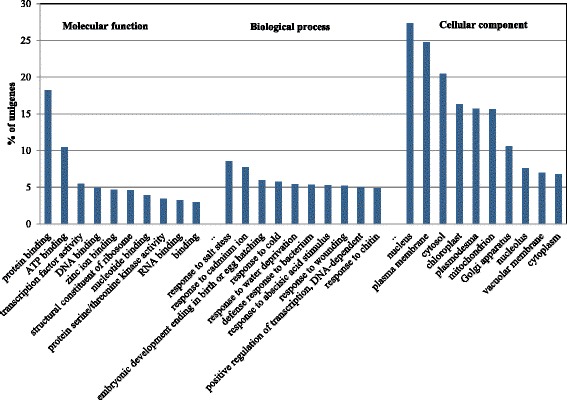
GO annotation of root transcripts in *P. ginseng*. A total of 41,244 transcripts in the Nr unigene set were assigned to at least one GO term in three categories, i.e., biological process, molecular function, and cellular component

**Fig. 2:**
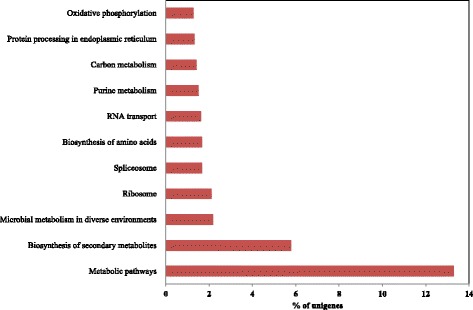
KEGG pathway assignments of *P. ginseng* root transcripts. Among Nr unigene sequences, 5,720 transcripts were assigned to KEGG pathways by KASS analysis. The percentage of the 5,720 transcripts assigned to the indicated pathways is shown

Additionally, GO terms were assigned to transcripts in the Nr unigene set based on their sequence matches to known protein sequences in the NCBI Nr database. A total of 41,244 (73.7 %) transcripts were assigned at least one GO term, including 37,435 (66.9 %) with terms in the biological process category, 32,995 (58.9 %) in the molecular function category, and 38,576 (68.9 %) in the cellular component category. Cellular component accounted for the majority of assigned GO terms, followed by the biological process and molecular function categories. Protein binding was the most abundant GO term within the molecular function category (Fig. 1). For the biological process category, response to salt stress and cadmium ion were the most highly represented terms (Fig. 1). Nucleus and plasma membrane were most abundant among various terms in the cellular component category. For further annotation, KEGG orthology (KO) categories were assigned to the transcripts. A total of 5,720 transcripts were assigned to 327 pathways, most of which were found in metabolic pathways (13.3 % of assigned transcripts) and biosynthesis pathways of secondary metabolites (5.7 % of assigned transcripts; Fig. 2).

### Expression profiling of unigenes

After the RNA reads were mapped onto transcript sequences of the Nr unigene set, transcript abundance (which represents expression) was determined by FPKM calculation. We investigated the 1,000 most abundant transcripts in the four root samples (Additional file 4: Table S1 and Additional file 5: Figure S3). The most abundant transcripts in one-year-old whole roots were those encoding ribonuclease, sucrose synthase, beta-amylase, major latex-like protein, heat shock proteins, and peroxidase. In tissues of six-year-old roots, the most abundant transcripts showed similarity to transcripts encoding metallothionein protein (MT), auxin-repressed protein (DRM1), glucose-1-phosphate adenylyltransferase, major latex-like protein, phloem protein 2, and heat shock protein.

### Investigation of differentially expressed transcripts among root samples

**Fig. 3:**
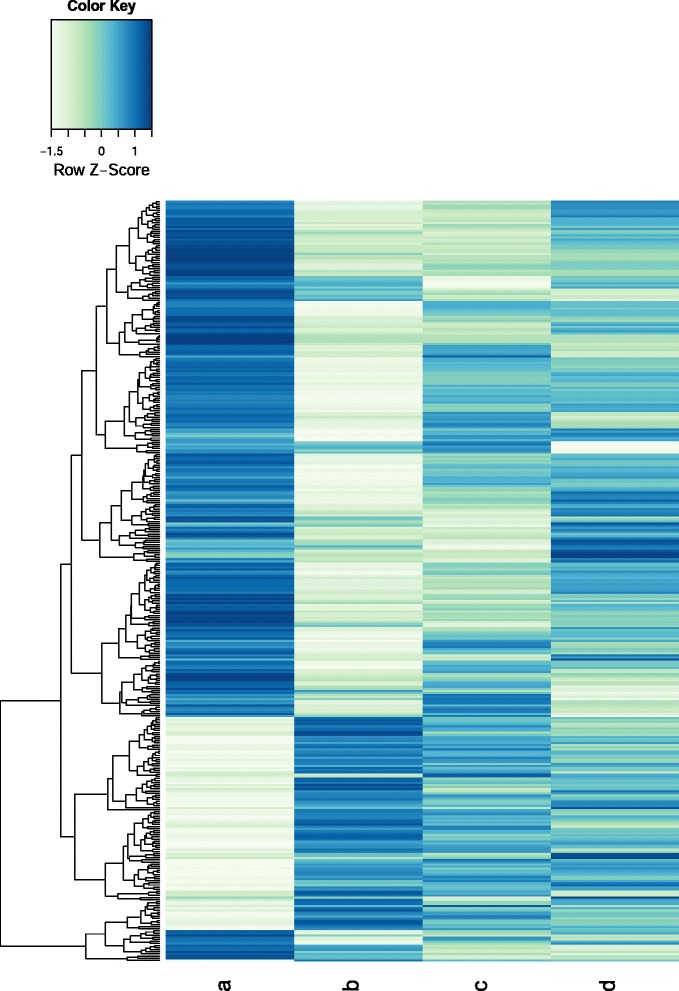
Expression profiles of differentially expressed transcripts among root samples. A total of 364 transcripts were identified to be differentially expressed among four root samples (**a** to **d**) using the edgeR Bioconductor package based on individual FPKM values of three biological replicates for each root sample. Heatmap shows the hierarchical clustering of average FPKM values obtained from individual FPKM values of three replicates. A indicates one-year-old whole roots, and **b**, **c**, and **d** represent main bodies, lateral roots, and rhizomes of six-year-old root samples, respectively

**Fig. 4:**
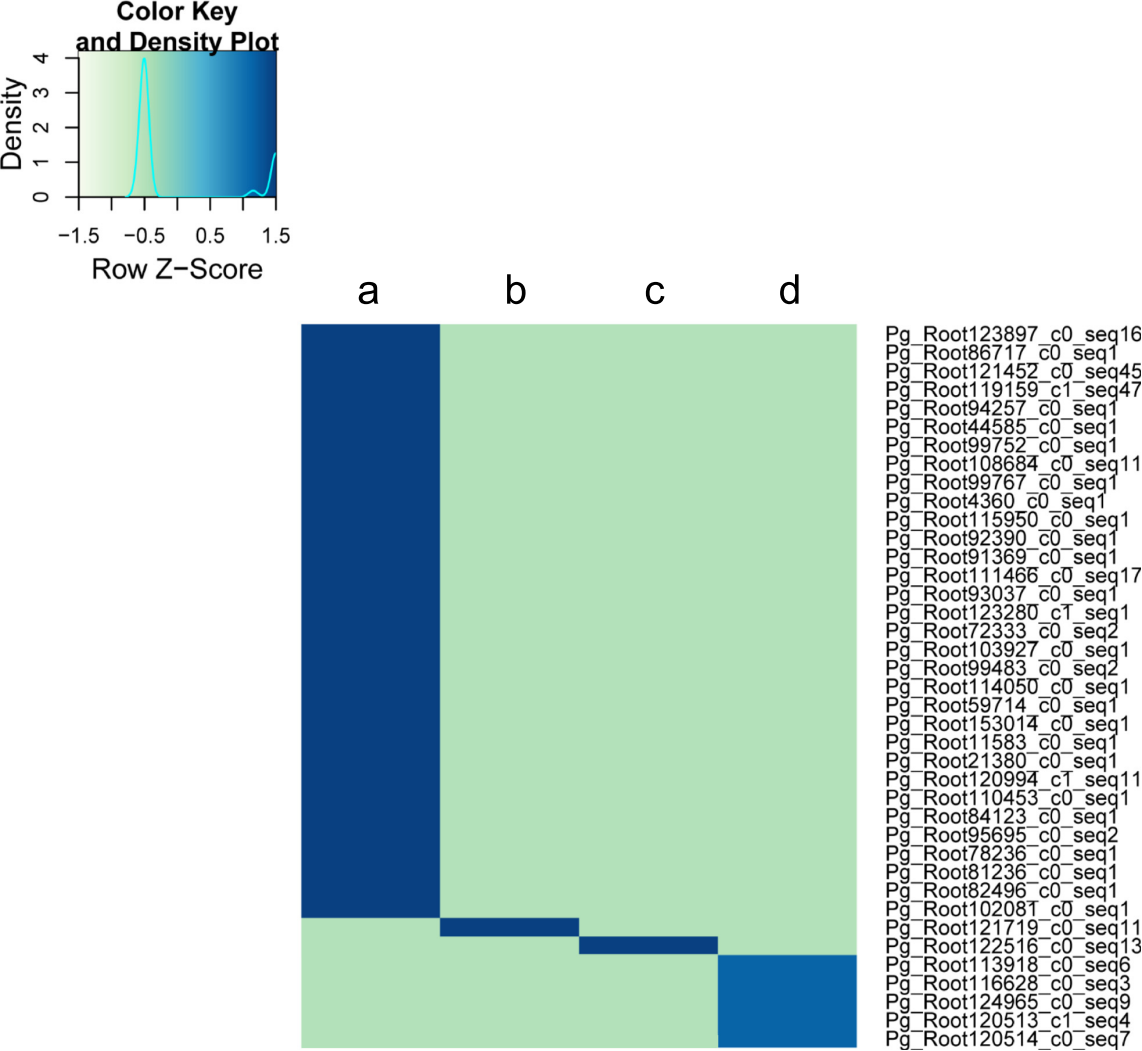
Expression profiles of specifically expressed transcripts in each of four root samples. A total of 39 transcripts were found to be specifically expressed based on the criteria of FPKM value > 3 in one sample and <1 FPKM in the other samples. Heatmap shows the hierarchical clustering of average FPKM values obtained from individual FPKM values of three replicates. **a** indicates one-year-old whole roots, and **b**, **c**, and **d** represent main bodies, lateral roots, and rhizomes of six-year-old root samples, respectively. Transcript IDs are shown to the right of the heatmap

To investigate differentially expressed (DE) transcripts among root samples, a statistical method using edgeR was applied. A total of 364 transcripts were differentially expressed (with more than two-fold change) among root samples (Fig. 3, Additional file 4: Table S1). Based on KEGG pathway and GO annotation, we determined that most DE transcripts were related to starch and sucrose metabolism (KEGG pathway assignment) as well as root hair elongation, response to abscisic acid stimulus, and Golgi organization and biogenesis (GO terms annotation; Additional file 6: Figure S4). Furthermore, by comparing FPKM values among the four root samples, we determined that 39 transcripts were specifically expressed in a single root sample (Fig. 4, Additional file 4: Table S1). Among these, 32 transcripts were uniquely expressed in one-year-old roots, while seven were uniquely expressed in one of three tissues of six-year-old roots, including, one, one, and five uniquely expressed transcripts in the main root body, lateral roots, and rhizomes, respectively. GO annotation revealed that most of these specific transcripts are involved in binding, such as ATP binding, receptor binding, RNA binding, and nucleotide binding, as well as transporter- and translation-related biological processes.

### Identification of candidate genes involved in ginsenoside biosynthesis

**Fig. 5:**
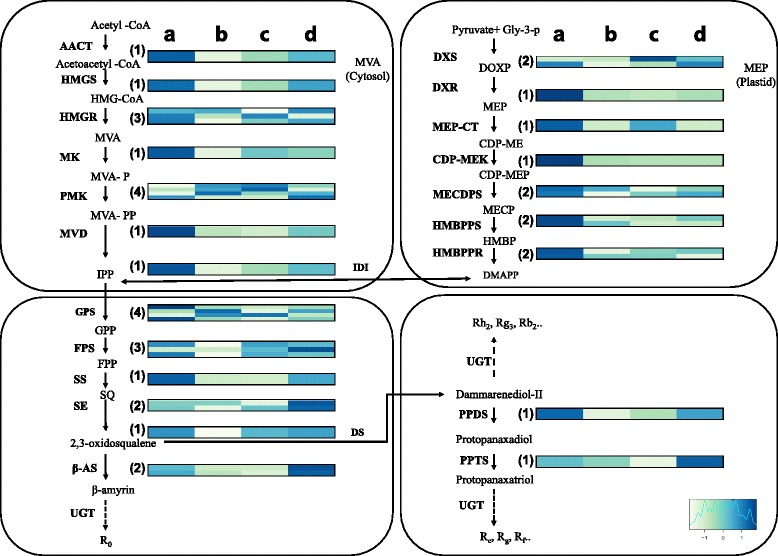
Expression profiles of transcripts encoding enzymes involved in ginsenoside biosynthesis in *P. ginseng*. Enzymes functioning in each step of the MVA and MEP pathways are indicated by bold letters. The expression patterns of transcripts encoding these enzymes are shown in the heatmap, which was constructed using average FPKM values obtained from individual FPKM values of three replicates. **a** indicates one-year-old whole roots and **b**, **c**, and **d** represent main bodies, lateral roots, and rhizomes of six-year-old root samples, respectively. The number of transcripts identified is shown in parentheses to the left of heatmap. AACT, acetyl CoA acetyltransferase; Beta-AS, beta-amyrin synthase; CDP-MEK, 4-diphosphocytidyl-2-C-methyl-D-erythritol kinase; DS, dammarenediol II synthase; DXR, 1-deoxy-D-xylulose-5-phosphate reductoisomerase; FPS, farnesyl diphosphate synthase; GPS, geranylgeranyl diphosphate synthase; HMBPPR, 4-hydroxy-3-methylbut-2-enyl diphosphate reductase; HMBPPS, 2-C-methyl-D-erythritol 2,4-cyclodiphosphate synthase; HMGR, 3-hydroxy-3-methylglutaryl-coenzymeA reductase; HMGS, hydroxymethyl glutaryl CoA synthase; IDI, isopentenyl-diphosphate delta-isomerase; MECDPS, 2-C-methyl-D-erythritol 2,4-cyclodiphosphate synthase; MEP-CT, 2-C-methyl-D-erythritol4-phosphate cytidylyltransferase; MK, mevalonate kinase; MVD, mevalonate diphosphate decarboxylase; PMK, phosphomevalonate kinase; PPDS, protopanaxadiol synthase; PPTS, protopanaxatriol synthase; SE, squalene monooxygenase/epoxidase; SS, squalene synthase

During ginsenoside biosynthesis, the precursor for terpenoid backbone production is oxidosqualene, which is biosynthesized via the mevalonate (MVA) and 2-C-methyl-D-erythritol-4-phosphate (MEP) pathways [24]. Based on KEGG assignments, a total of 38 transcripts encoding enzymes involved in ginsenoside biosynthesis via both the MVA and MEP pathways were identified (Fig. 5, Additional file 7: Table S2). Among these, 26 genes were present in multiple copies (two, three, and four copies for six, two, and two genes, respectively), which might have been derived from genome or gene duplication. We also identified candidates for major downstream genes such as those encoding farnesyl diphosphate synthase (FPS), squalene epoxidase (SE), and dammarenediol II synthase (DS). Furthermore, two CYP450 genes encoding protopanaxadiol synthase (CYP716A47) and protopanaxatriol synthase (CYP716A53V2, involved in dammarane-type ginsenoside biosynthesis) [25, 26] were found in our unigene set.

Most transcripts in this pathway were more highly expressed in one-year-old whole roots than in tissues of six-year-old roots, while some transcripts were more highly expressed in six-year-old root tissues, such as genes encoding SE, beta-amyrine synthase (β-AS), and protopanaxatriol synthase (PPTS), which function in downstream steps of the ginsenoside biosynthesis pathway (Fig. 5). In six-year-old roots, the transcripts were strongly expressed in rhizomes, followed by lateral roots and main root bodies. Only transcripts encoding 3-hydroxy-3-methylglutaryl-coenzymeA reductase (HMGR), phosphomevalonate kinase (PMK), and geranylgeranyl diphosphate synthase (GPS) were highly expressed in main root bodies. Overall, the expression tendency of the transcripts was similar between the upstream steps of the two pathways. Transcripts encoding HMGR and 1-deoxy-D-xylulose-5-phosphate synthase (DXS) were also identified as DE genes between one- and six-year-old main root bodies and rhizomes.

Glycosylation, the last step in ginsenoside biosynthesis, is catalyzed by UDP glycosyltransferase (UGT). UGTs containing plant secondary product glycosyltransferase (PSPG) motif sequences are involved in the glycosylation of plant secondary metabolites [27, 28]. Accordingly, we collected PSPG motif-containing consensus UGT sequences from PROSITE (http://prosite.expasy.org/, Accession no. PS00375) and searched against the Nr unigene set using BLASTX. A total of 184 transcripts were identified in the Nr unigene set. The abundance of these UGT transcripts was highly variable among the four root samples, and therefore represented strongly biased expression patterns (Additional file 8: Figure S5). To identify strong candidate UGT transcripts that function in downstream steps of the ginsenoside biosynthesis pathway, we performed co-expression analysis of the 184 transcripts. However, we failed to detect UGT transcripts that were co-expressed with the 38 transcripts putatively involved in ginsenoside biosynthesis pathways.

## Discussion

### Comprehensive root transcriptome profiling in *P. ginseng*

*P. ginseng* is a highly therapeutic nutraceutical herbal plant containing ginsenosides with various pharmacological activities. Among ginseng plant parts, roots are the primary tissue used for commercial production [21]. Large-scale transcriptome sequencing allows us to investigate gene expression and to perform various functional genomic studies. RNA-seq, an NGS methodology for RNA profiling, is currently the best method for reconstructing the whole transcriptome and for profiling gene expression at unprecedented resolution [29, 30]. RNA-seq produces huge and complex data sets with some technical issues such as sequencing errors and biases due to amplification and fragmentation during various steps of the RNA-seq protocol [31]. Thus, deriving biologically meaningful findings from such a large, complex data set remains quite challenging. Furthermore, it is not always possible to uniquely align short sequencing reads to a gene, as genomes contain numerous repeats and paralogous genes with high sequence similarity. Therefore, in this study, we applied a computational pipeline to reduce the complexity and to enhance the biologically meaningful interpretation of our RNA-seq data (Additional file 1: Figure S1).

Prior to this study, Li *et al.* [15] generated cDNA libraries from four tissues (root, stems, leaves, and flowers) of ginseng, which are assumed to represent an updated set of unigenes for *P. ginseng*. Although they were assembled from different tissues, over 89 % of those previously reported unigenes were present in our Nr assembly set. Furthermore, we identified a large number of novel transcripts, accounting for 28.6 % of our Nr unigene set. The results indicate that our transcriptome data include many *P. ginseng* genes beyond those reported in this plant species to date, even though our data were generated only from root tissues. These numerous novel transcripts provide exciting opportunities for further study of functional genomics in *P. ginseng*. We also functionally annotated over 90 % of the unigenes in our set, highlighting the accuracy of our approach and of our assembled unigene set. This annotation rate is much higher than those reported for other ginsengs, such as *P. notoginseng* and *P. quinquefolius*, as well as for previous *P. ginseng* transcriptome studies [15, 32–34]. While we assume that we obtained an almost complete gene set for root tissues, more comprehensive transcriptome data from various other tissues will be necessary to obtain a complete gene set for *P. ginseng*.

### Expression profiling of the root transcriptome

In previous ginseng transcriptome studies, the most abundant genes were related to ribonuclease, major latex-like protein, anti-oxidant enzymes, and sugar and energy metabolic pathways. Such genes were also identified among the most abundant transcripts in the current study (Additional file 4: Table S1). In particular, transcripts encoding MT and DRM1 were the most abundant in main root bodies, lateral roots, and rhizomes of six-year-old roots. The *MT* gene is also abundantly represented in four-year-old *P. ginseng* root [22] and *P. quinquefolius* leaf EST libraries [35]. *MT* primarily functions in metal homeostasis and abiotic stress responses [36]. Additionally, *MT* genes are involved in root development and protection against reactive oxygen species [37]. Similarly, auxin-repressed gene transcripts are abundant among in *P. quinquefolius* root ESTs [35, 38]. Auxin-repressed genes such as *DRM1* enable plants to respond to stress to enhance their survival, and they are also highly expressed in mature tissues [39, 40]. Since ginseng has a long life span, it is frequently exposed to unfavorable environments and various stresses. Thus, our results imply that the abundance of *MT* and *DRM1* transcripts help protect the plant from various environmental stresses.

We identified a set of DE transcripts among root samples using high throughput replicated sequence data. Tani *et al*. [41] reported that carbohydrates are the major components of one- to two-year-old roots compared to six-year-old roots. Notably, we also detected genes involved in sugar metabolism as more highly expressed in one-year-old roots compared to six-year-old roots and rhizomes (Additional file 6: Figure S4). Rhomboid-like protein (RBL) is required for root growth and floral development, and it exhibits differential expression in specific tissues during different developmental stages in *A. thaliana* and other eukaryotes [42]. We found that the most highly expressed specific gene, Pg_Root111466_c0_seq17, in one-year-old roots encodes RBL (Additional file 4: Table S1), suggesting that RBL plays a vital role in root development in one-year-old *P. ginseng* plants. Similarly, the most highly expressed specific gene, Pg_Root120514_c0_seq7, in six-year-old rhizomes encodes gibberellin-regulated protein (Additional file 4: Table S1), which is also encoded by a rhizome-specific gene in *Oryza longistaminata* [43].

### Genes involved in ginsenoside biosynthesis

Chen *et al.* [14] and Li *et al.* [15] sequenced the transcriptomes of 11-year-old and four-year-old roots of *P. ginseng*, respectively, revealing many genes involved in ginsenoside biosynthesis. Nonetheless, the β-AS gene and MEP pathway genes, which also contribute to ginsenoside biosynthesis [24], have not previously been identified in the root transcriptome of *P. ginseng*. In this study, we identified candidate transcripts involved in ginsenoside biosynthesis, along with their expression profiles. The ginsenoside contents are highest in five- and six-year-old rhizomes, followed by lateral roots and the main root body [44–46]. The gene expression patterns obtained in the current study are in good agreement with these phenomena (Fig. 5). On the whole, the upstream ginsenoside pathway genes (including those encoding MEP and MVA) exhibited higher expression in one-year-old roots, while the downstream genes exhibited higher expression in six-year-old roots. This result suggests that upstream genes are more active in one-year-old roots (perhaps to produce several types of MEP- and MVA-derived primary and secondary metabolites rather than ginsenosides) compared to six-year-old roots. Among the putative pathway genes, *HMGR* and *DXS* genes exhibited significant differential expression between roots and rhizomes. We identified three *HMGR* transcripts based on KEGG annotation, one of which was more highly expressed in the main root body than in lateral roots, as observed by Kim *et al.* [47]. Previously, four candidate *UGT* genes were identified in *P. quinquefolius* [33], all of which were identified among six unigenes in *P. ginseng* using homology searches [15]. Recently, 12 *UGT* genes were identified and characterized by Khorolragchaa *et al.* [28] from ESTs of *P. ginseng*. In the current study, we identified a set of *UGT* genes including previously identified unigenes in *P. quinquefolius* and *P. ginseng.*

## Conclusions

This study provides large-scale root transcriptome data for Korean ginseng using a newly designed assembly method*.* This genetic resource will help provide new insights into the roles of genes in development and secondary metabolite biosynthesis in Korean ginseng and other plant species.

## Methods

### Plant materials and RNA isolation

One- and six-year-old roots of *P. ginseng* cv. Chunpoong (ChP), a genetically inbred line, was utilized for ginseng genome sequencing [1]. One-year-old roots were collected from plants with fully expanded leaves grown in a growth chamber (24 °C, 60 % relative humidity, 16-h day length, and light intensity of 40 μmol m^−2^ s^−1^). Six-year-old roots were collected from plants with fully expanded leaves grown in a ginseng experimental field (Suwon, Korea), which were divided into three parts, including the main root body, lateral roots (including fine roots), and rhizomes. The samples were immediately frozen in liquid nitrogen and stored at −80 °C until use. Three independent biological replicates were prepared and each replicate included root materials from three or more of *P. ginseng* plants. Total RNA was isolated using a Plant RNeasy mini kit (QIAGEN, Germany) and/or Hybrid-R kit (GeneAll, Korea) according to the manufacturers’ instructions. Approximately 2 μg total RNA was used for RNA-seq library construction after examination of its quality and quantity using a Bioanalyzer (Agilent Technologies, USA).

### Illumina sequencing and quality control

RNA-seq libraries with an insert size of 300 bp were prepared independently for three biological replicates of four root samples using an Illumina TruSeq RNA Sample Preparation Kit according to the manufacturer’s instructions. Libraries from the first replicate were sequenced using the Illumina HiSeq2000 platform with paired-end (PE) reads of 101 bp at Macrogen Co. (Seoul, Korea), while the remaining replicates were sequenced using the Illumina NextSeq 500 platform with a PE read length of 150 bp at LabGenomics Co. (Pankyo, Korea). A stringent quality control process was carried out to filter high-quality RNA reads and to discard reads with adaptor contamination using an NGS QC Toolkit (v2.3.3) [48].

### *De novo* assembly and annotation

*De novo* assembly was carried out using Trinity (trinityrnaseq_r20140413) with default parameters [49]. Subsequently, to obtain a non-redundant (Nr) unigene set, all contaminating transcripts were removed by aligning the reads against the microbial genome database (http://mbgd.genome.ad.jp/). Ribosomal RNA (rRNA) sequences were then removed by predicting rRNA sequences using RNAmmer (v1.2) [50] and aligning them against an rRNA database [51]. Long noncoding RNAs were eliminated using the approach developed by Li *et al*. [52]. To determine genomic locations, all filtered transcripts were aligned onto the newest ginseng scaffold sequences [23] using GMAP (version 2013-10-28) with the parameter of --min-intronlength = 10, −K = 10,000 and --min-identity = 95.0 [53]. Finally, in-house Perl and Python scripts were used to cluster the transcripts based on the gene loci and the selected consensus structure, respectively (Additional file 1: Figure S1). Among the final consensus clusters, a unigene sequence was selected from each cluster based on sequence length. Furthermore, using previously described methods [17], read-depth analysis of unaligned transcripts was performed, and a non-redundant set of transcripts was generated based on sequence length; these transcripts were combined to form the final Nr unigene set.

### Functional annotation

To investigate the putative function of each transcript in the Nr unigene set, Gene Ontology (GO) analysis was performed using Blast2GO [54]. First, the transcripts were annotated against the Nr protein database downloaded from NCBI using local BLASTX with an E-value threshold of 10^−3^. Based on the annotation information, GO terms in three categories (molecular function, biological process, and cellular component) were assigned to transcripts using Blast2GO. Metabolic pathway mapping of transcripts in the Nr unigene set was performed using the KEGG Automatic Annotation Server (KAAS; http://www.genome.jp/tools/kaas/) [55].

### Expression profiling

High-quality RNA-seq reads obtained from filtering were aligned to the transcripts to estimate the transcript abundance using RSEM (v1.2.4) (RNA-Seq by Expectation Maximization) with the parameter of minimum and maximum fragment length of 200 and 300 respectively [56]. RSEM calculates the number of RNA reads or fragments mapped to transcripts as FPKM (Fragments Per Kilobase per Million) values. To identify differentially expressed transcripts among the four samples, the Bioconductor package edgeR [57] was used. Transcripts that had significant false discovery rate (FDR) values of up to 0.01 and fold change values greater than 2 were considered to be differentially expressed. To identify transcripts that were specifically expressed in a single sample, FPKM values were compared among samples, and transcripts with FPKM >3 in a single tissue and FPKM <1 in the other three tissues were selected.

### Availability of supporting data

The Illumina RNA-seq data generated from root tissues of *Panax ginseng* are available in the NCBI SRA with accessions SRR1648364, SRR1649308, and SRR1649311 for three replicates of one-year-old whole roots; SRR1648377, SRR1649321, and SRR1649325 for three replicates of lateral roots; SRR1648366, SRR1649315, and SRR1649319 for three replicates of main root bodies; SRR1648380, SRR1649327, and SRR1649331 for three replicates of rhizomes. All assembled transcripts and their analysis results are available at http://im-crop.snu.ac.kr/transdb/data.php.

## Additional files

Additional file 1: Figure S1. Pipeline for selection of the Nr unigene set from *de novo* assembled transcriptome data. Additional file 2: Figure S2. Length distribution of transcripts in the Nr unigene set. Additional file 3: Dataset S1. A compressed file containing all assembled root transcripts and their functional annotations from BLASTX search. Additional file 4: Table S1. List of genes identified to be the most abundantly, differentially, and specifically expressed in root samples. Additional file 5: Figure S3. Expression profile of the 1,000 most abundant transcripts in four root samples of *P. ginseng*. Heatmap shows the hierarchical clustering of average FPKM values obtained from individual FPKM values from three replicates. A indicates one-year-old whole roots, and B, C, and D represent main bodies, lateral roots, and rhizomes of six-year-old roots, respectively. Additional file 6: Figure S4. GO analysis and KEGG pathway assignments of differentially expressed (DE) transcripts among four root samples of *P. ginseng*. (A) All 364 DE transcripts were assigned to at least one GO term in three categories. (B) 192 DE transcripts were assigned to KEGG pathways by KASS analysis. The x-axis indicates the number of DE transcripts assigned to each pathway (listed on the y-axis). Additional file 7: Table S2. List of candidate transcripts involved in ginsenoside biosynthesis and their expression profiles in root samples. Additional file 8: Figure S5. Expression profiles of 184 UGT genes identified in the Nr unigene set. Heatmap shows the hierarchical clustering of average FPKM values obtained from individual FPKM values from three replicates. A indicates one-year-old whole roots, and B, C, and D represent main bodies, lateral roots, and rhizomes of six-year-old roots, respectively.

## Abbreviations

bp: Base pair
kb: Kilobase pair
FPKM: Fragments per kilobase of exon per million fragments
ORF: Open reading frame
NGS: Next generation sequencing
MEP: 2-C-methyl-D-erythritol-4-phosphate
MVA: Mevalonate
KEGG: Kyoto encyclopedia of genes and genomes
EC: Enzyme commission
