# Supplementary figures and images for: Comprehensive analysis of Panax ginseng root transcriptomes
Source: BMC Plant Biol. 2015 Jun 12;15:138. doi: 10.1186/s12870-015-0527-0 (PMC4464628; doi:10.1186/s12870-015-0527-0)

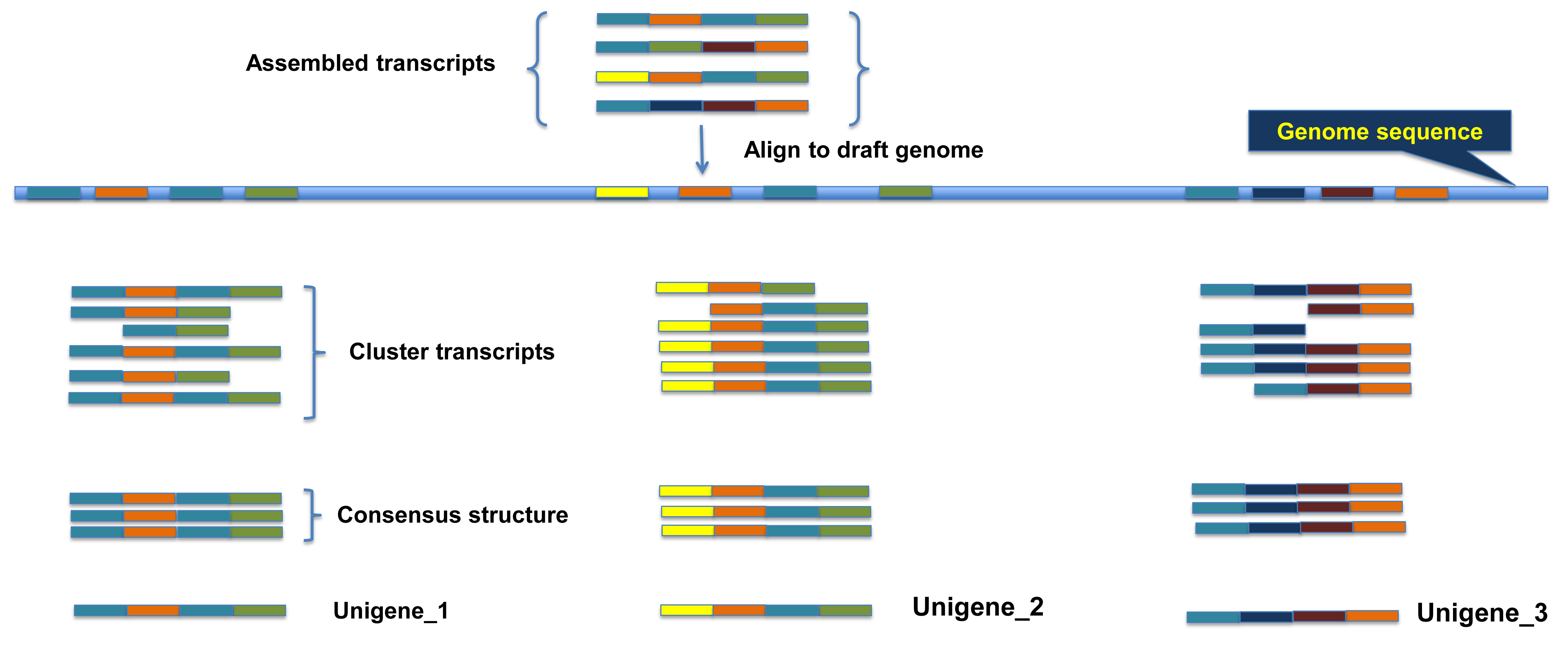

Supplement: Additional file 1: Figure S1. — Pipeline for selection of the Nr unigene set from de novo assembled transcriptome data. [file 12870_2015_527_MOESM1_ESM.tif]

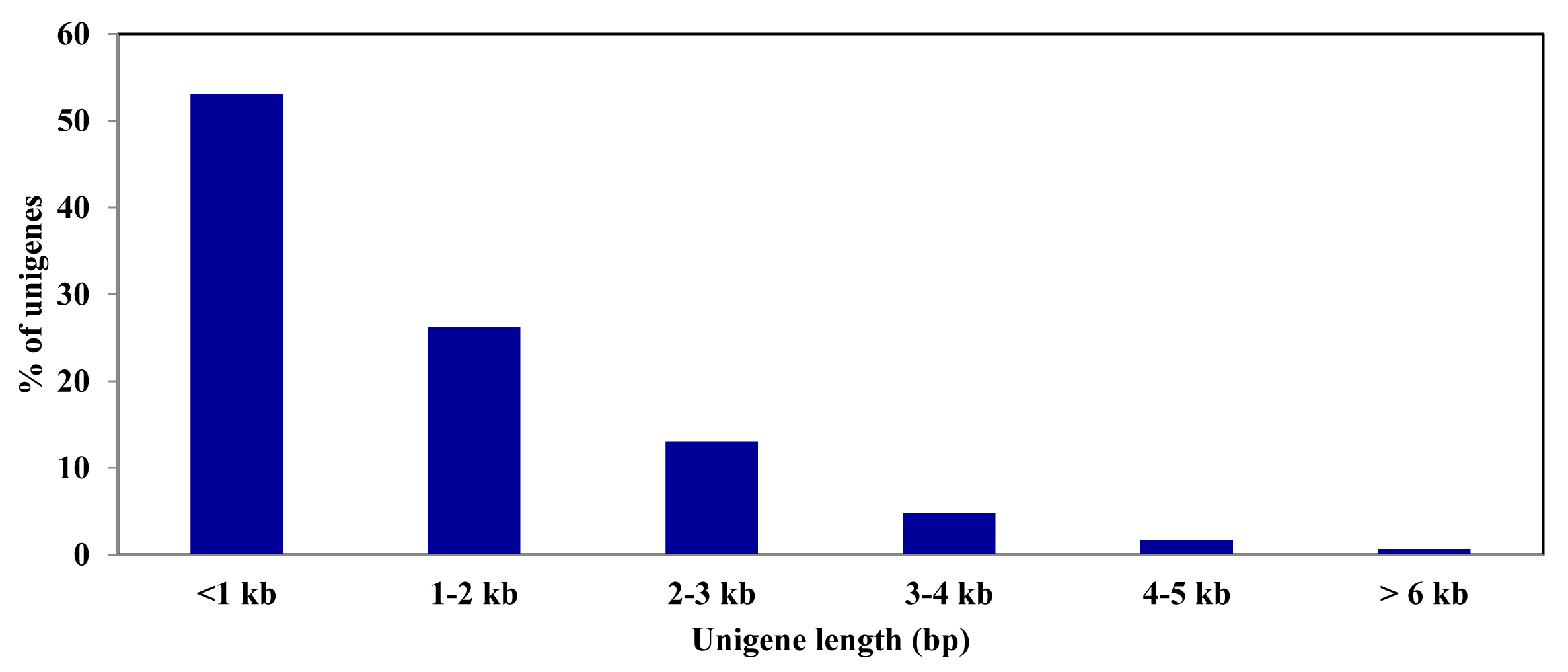

Supplement: Additional file 2: Figure S2. — Length distribution of transcripts in the Nr unigene set. [file 12870_2015_527_MOESM2_ESM.tif]

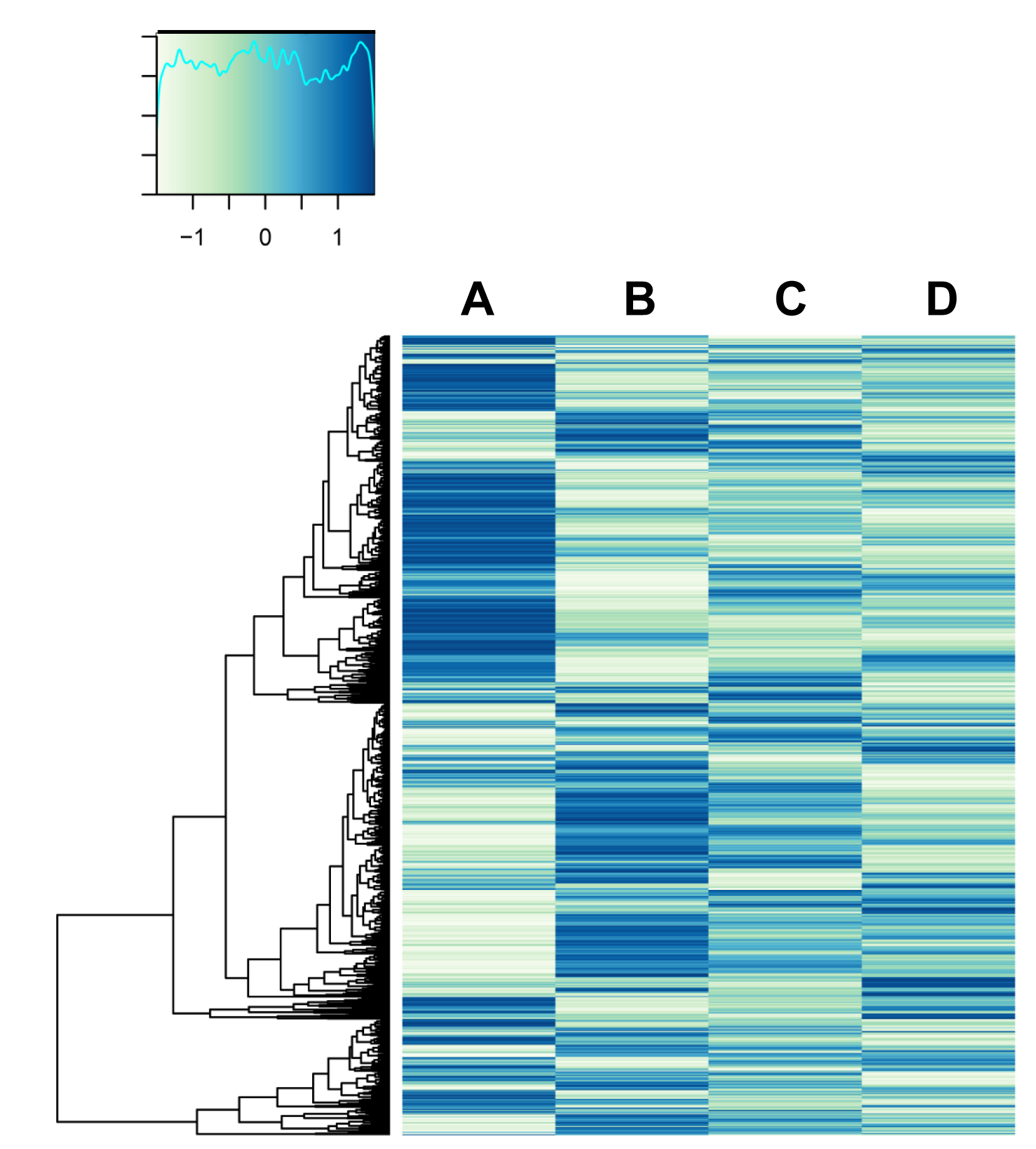

Supplement: Additional file 5: Figure S3. — Expression profile of the 1,000 most abundant transcripts in four root samples of P. ginseng. Heatmap shows the hierarchical clustering of average FPKM values obtained from individual FPKM values from three replicates. A indicates one-year-old whole roots, and B, C, and D represent main bodies, lateral roots, and rhizomes of six-year-old roots, respectively. [file 12870_2015_527_MOESM5_ESM.tif]

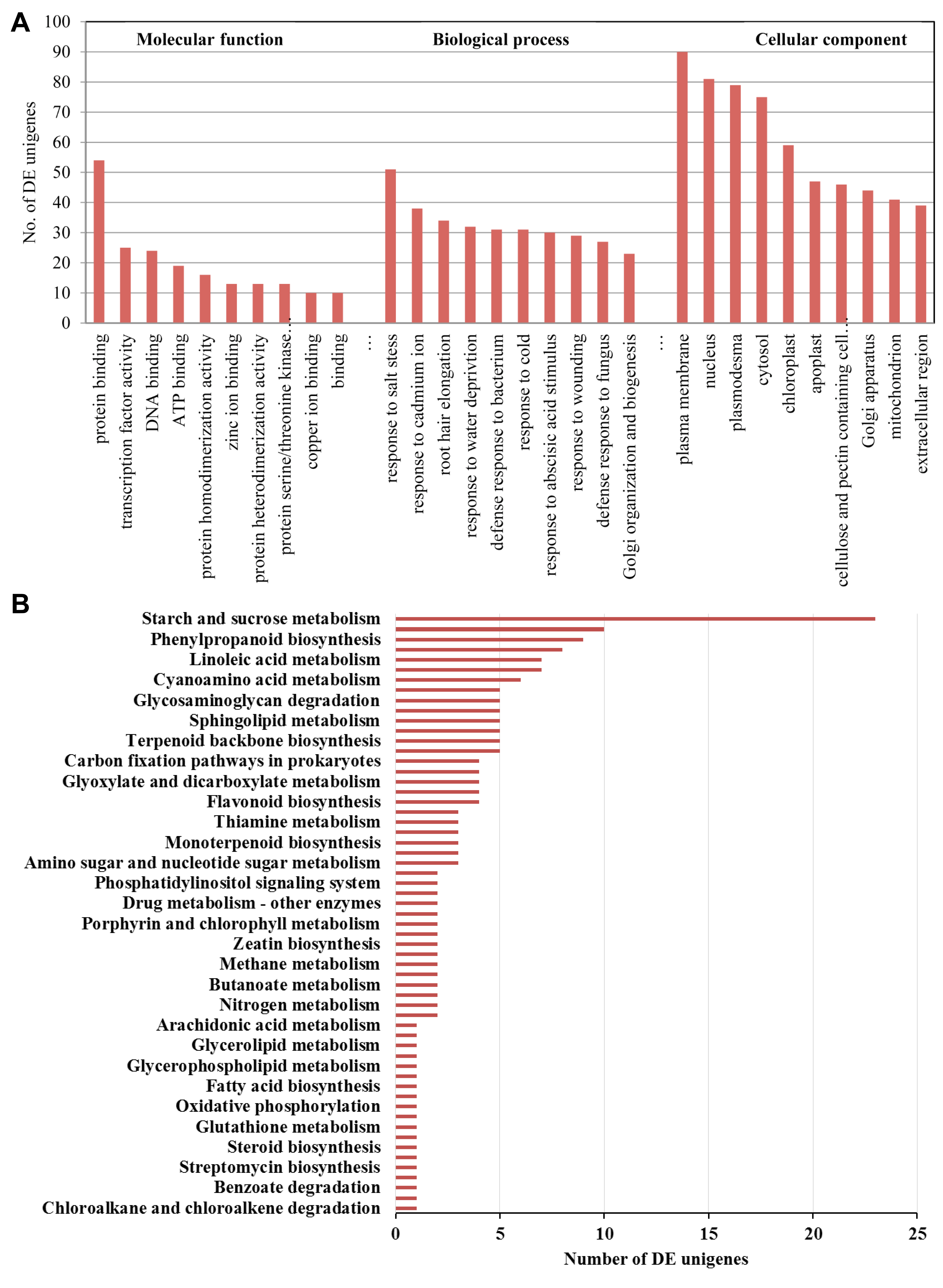

Supplement: Additional file 6: Figure S4. — GO analysis and KEGG pathway assignments of differentially expressed (DE) transcripts among four root samples of P. ginseng. (A) All 364 DE transcripts were assigned to at least one GO term in three categories. (B) 192 DE transcripts were assigned to KEGG pathways by KASS analysis. The x-axis indicates the number of DE transcripts assigned to each pathway (listed on the y-axis). [file 12870_2015_527_MOESM6_ESM.tif]

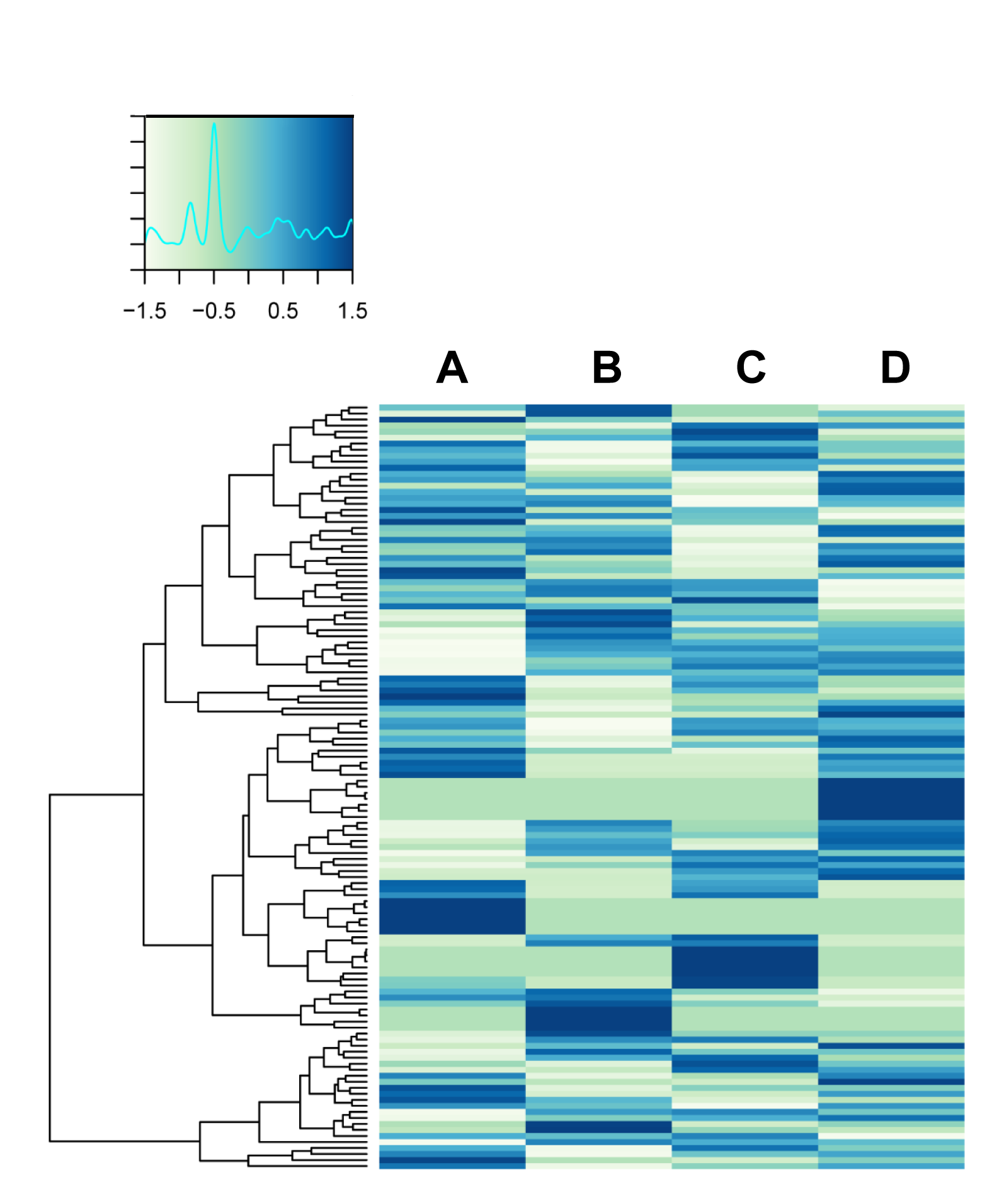

Supplement: Additional file 8: Figure S5. — Expression profiles of 184 UGT genes identified in the Nr unigene set. Heatmap shows the hierarchical clustering of average FPKM values obtained from individual FPKM values from three replicates. A indicates one-year-old whole roots, and B, C, and D represent main bodies, lateral roots, and rhizomes of six-year-old roots, respectively. [file 12870_2015_527_MOESM8_ESM.tif]
